# Supplementary material for: 6-Gingerol Ameliorates Hepatic Steatosis, Inflammation and Oxidative Stress in High-Fat Diet-Fed Mice through Activating LKB1/AMPK Signaling
Source: Int J Mol Sci. 2023 Mar 27;24(7):6285. doi: 10.3390/ijms24076285 (PMC10094681; doi:10.3390/ijms24076285)
Supplement: Supplementary file 1 [file ijms-24-06285-s001.zip › ijms-2261220-supplementary.pdf]

Supplementary Materials for

6-Gingerol Ameliorates Hepatic Steatosis and Oxidative Stress in High-Fat Diet-Fed Mice through Strengthening LKB1/STRAD/MO25 Complex Stability to activate LKB1/AMPK Pathway

Table S1. Primers used in RT-qPCR analysis.

Gene	Forward primer (5’→3’)	Reverse primer (5’→3’)
Acaca	TGAAGCTGGACCTAGAAGAGA	AGGCCAAACCATCCTGTAAG
Actb	GTGCTATGTTGCTCTAGACTTCG	ATGCCACAGGATTCCATACC
Cpt1a	CTGCACTCCTGGAAGAAGAAG	CACCCACCACCACGATAAG
Fasn	CTCATTGGTGGTGTGTGGACAT	TTGGAGAGATCCTTCAGCTTTC
Ppara	CTGTCGGGATGTCACACAAT	CAGGTCGTGTTCACAGGTAAG
Pgc1a	CCCAGATCTTCCTGAACTTGAC	ATTGGTCGCTACACCACTTC
Scd1	AGAAGACATCCGTCCTGAAATG	CAGCAGGACCATGAGAATGAT
Srebf1c	AGAAGACATCCGTCCTGAAATG	CAGCAGGACCATGAGAATGAT

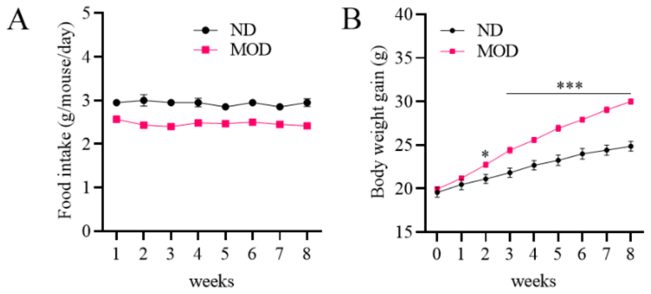

Figure S1. (A) Food intake and (B) Body weight gain during modeling (recorded once a week). $^{*}p < 0.05$ and $^{***}p < 0.001$, compared with the ND group.

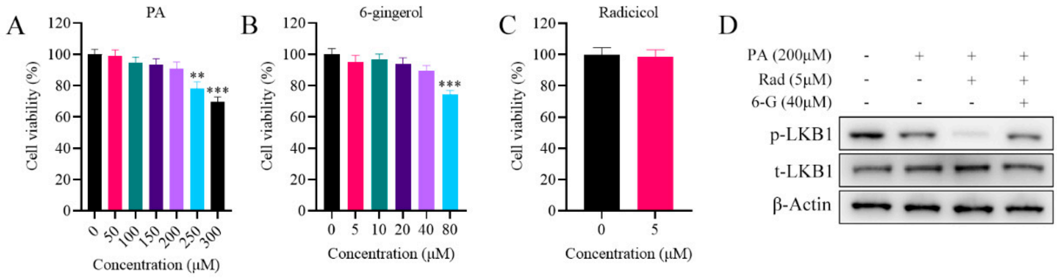

Figure S2. Cell viability under treatments with different concentrations of (A) PA (0μM, 50μM, 100μM, 150μM, 200μM, 250μM, 300μM) and (B) 6-gingerol (0μM, 5μM, 10μM, 20μM, 40μM and 80μM) and (C) 5μM Radicicol. Mean ± SEM. (D) Protein expressions of p-LKB1 and t-LKB1 in HepG2 cells by western blotting. $^{**}p < 0.01$ and $^{***}p < 0.001$, compared with the 0μM group.
